# The impact of health‐policy‐driven subsidisation of prostate magnetic resonance imaging on transperineal prostate biopsy practice and outcomes

**DOI:** 10.1002/bco2.140

**Published:** 2022-02-11

**Authors:** Gavin Wei, Fairleigh Reeves, Marlon Perera, Brian D. Kelly, Stephen Esler, Damien Bolton, Greg Jack

**Affiliations:** ^1^ Department of Surgery Austin Health, The University of Melbourne Melbourne Victoria Australia; ^2^ Olivia Newton‐John Cancer and Wellness Centre Austin Health Heidelberg Victoria Australia; ^3^ Department of Urology Memorial Sloan Kettering Cancer Center New York New York USA; ^4^ Department of Radiology Austin Health, The University of Melbourne Melbourne Victoria Australia

**Keywords:** diagnosis, elevated PSA, MRI, prostate cancer, public health

## Abstract

**Background:**

From 1 July 2018, the Australian Medicare Benefits Schedule (MBS) introduced rebates for multi‐parametric magnetic resonance imaging (mpMRI) for the workup for prostate cancer (PCa). We aimed to determine if subsidisation of mpMRI prior to transperineal biopsy altered our institution's prostate biopsy practice patterns and outcomes.

**Methods:**

All patients who underwent transperineal prostate biopsy at an Australian tertiary institution from 1 January 2017 to 1 January 2020 were identified. Patients with known PCa were excluded. Patients were stratified into two groups: a pre‐subsidisation cohort comprising patients biopsied prior to the introduction of mpMRI subsidisation on 1 July 2018 and a post‐subsidisation cohort comprising patients biopsied after 1 July 2018. Histopathological results were compared with further stratification based on mpMRI results. Clinically significant cancer was defined as ISUP Grade Group ≥ 2.

**Results:**

Six hundred and fifty men fulfilled the inclusion criteria. Three hundred and sixty‐one patients were in the pre‐subsidisation cohort and 289 in the post‐subsidisation cohort. Of the patients in the pre‐subsidisation group, 36.3% underwent a pre‐biopsy mpMRI compared with 77.5% in the post‐subsidisation group. Of the patients in the pre‐subsidisation group, 59.6% had positive biopsies (*p* = 0.024) compared with 68.2% in the post‐subsidisation group. The rate of clinically significant PCa was lower in the pre‐subsidisation group (39.1%) compared with the post‐subsidisation (49.5%, *p* = 0.008). The negative predictive value of mpMRI for clinically significant PCa was 86.5%.

**Conclusion:**

Our institution experienced a reduction of negative prostate biopsies and an increase in clinically significant PCa within transperineal biopsy specimens after the Australian healthcare system introduced financial subsidisation of mpMRI.

## 1 INTRODUCTION

Prostate cancer (PCa) remains the second most frequently diagnosed cancer in men worldwide with almost 1.3 million new cases estimated in 2018.^1^ The decision to undertake prostate biopsy is often determined by a high prostate‐specific antigen (PSA) level or a suspicious digital rectal examination (DRE). Although this approach to cancer detection has seen a reduction in disease‐specific mortality, it has also resulted in many men undergoing negative biopsies and the increased detection of low‐grade, low‐risk PCa.^2^ Opponents of PCa screening argue that up to 42% of PCa may be overdiagnosed in Australia, placing some patients at risk of overtreatment and subjecting others to prolonged follow‐up with significant costs and burdens to the patient and healthcare system.^3^

Given the resources required and potential morbidity of biopsy, there is a great interest in optimising patient selection pre‐biopsy to reduce unnecessary biopsies. In recent years, multi‐parametric magnetic resonance imaging (mpMRI) of the prostate has become more widely available. The Prostate Imaging – Reporting and Data System (PI‐RADS) has enabled standardisation of mpMRI reporting worldwide.^4^ A growing body of evidence supports the role of pre‐diagnostic mpMRI in avoiding unnecessary prostate biopsy and assisting in identifying high risk lesions for targeting.^5^, ^6^, ^7^, ^8^ In addition, there are suggestions that mpMRI may help guide further management for patients with PCa.^9^, ^10^ Consequently, mpMRI has become increasingly utilised as a tool in the diagnosis of PCa.

Starting 1 July 2018, the financial burden of mpMRI of the prostate became subsidised by the Australian healthcare system for all patients fulfilling the Medicare Benefits Schedule (MBS) criteria.^11^ We aimed to determine the effects this has had on patient selection for prostate biopsy and biopsy outcomes at our institution.

## 2 METHODS

All patients who underwent transperineal prostate biopsy at a large, publicly funded, Australian tertiary institution between 1 January 2017 and 1 January 2020 were identified from a prospectively maintained database. Patients with known PCa, such as those on an active surveillance regimen, were excluded from the study. Approval for this project was granted by our institution's Human Research Ethics Committee.

Patients were stratified into two groups: the pre‐MBS subsidisation cohort encompassed all patients biopsied prior to 1 July 2018, whereas the post‐MBS subsidisation cohort comprised patients biopsied from 1 July 2018.

Following an initial consultation for elevated PSA, patients were counselled regarding the benefits and costs, where applicable, of mpMRI. Decision to obtain mpMRI was determined through shared decision making between the surgeon and patient based on clinical indications. mpMRI of the prostate was performed in Medicare approved imaging centres at 3 Tesla with diffusion weighted, dynamic contrast enhanced imaging. Standardised reporting was performed by prostate radiologists using the PI‐RADS version 2.^4^ For this study, a positive mpMRI was considered to be PI‐RADS ≥ 3. Prior to the introduction of the MBS rebate, patients were required to pay approximately AUD $400.00 for their mpMRI. Following MBS subsidisation, mpMRI was fully subsidised for all patients who met the MBS eligibility criteria (Item 63541K & 63542 NK).^11^ Eligibility criteria are outlined in Table S1.

Based on mpMRI findings and shared decision making with the patient, a decision to proceed with prostate biopsy was considered. Transperineal biopsy was performed by a urologist under general or sedated anaesthesia with the patient in low lithotomy position using a bi‐planar ultrasound transducer probe (BK Medical Holdings Ltd, Peabody, USA) in the rectum and an 18 g × 22 cm biopsy needle (Bard Max Core Needle, Bard, USA). Systematic biopsy of the entire prostate was performed in 5‐mm increments utilising a brachytherapy template grid (Accucare Template grid, Civco Medical Solutions, UK). All patients underwent systematic biopsy in addition to cognitively targeted biopsies from areas of concern identified by mpMRI. The template and total number of cores taken were in accordance with the Ginsburg protocol.^12^ Specimens were assessed using the International Society of Urological Pathology (ISUP) Grade Group system and were reviewed at a Urology multidisciplinary team meeting by dedicated uro‐pathologists.^13^ For this study, clinically significant cancer was defined as ISUP Grade Group ≥ 2.

Medical records were reviewed and data obtained included age, pre‐biopsy PSA, family history, history of previous biopsy, DRE findings, prostate volume, PI‐RADS score, histopathology results and duration from initial urological consultation to mpMRI and biopsy.

Chi‐square analysis and Mann–Whitney *U* test were employed for categorical and continuous variables respectively to provide comparisons between patients who underwent diagnostic transperineal prostate biopsies pre‐ and post‐government subsidised mpMRI. An *α* value of 0.05 was used to determine statistical significance. Statistical analyses were performed using SPSS, version 26.0 (IBM Corp., Armonk, NY, USA).

## 3 RESULTS

Six hundred and fifty men met the inclusion criteria. Three hundred and sixty‐one men were in the pre‐MBS subsidisation cohort, and 289 men were in the post‐MBS subsidisation cohort. Both cohorts were similar in age, clinical stage and prior biopsy history. The post‐subsidisation cohort had higher PSA (7.6 ng/ml vs. 6.6 ng/ml) and larger prostate volume (45 ml vs. 40 ml) compared with the pre‐subsidisation cohort. There was a longer time interval between initial urological consultation and prostate biopsy in the post‐subsidisation cohort compared with pre‐subsidisation cohort with a median of 63 and 45 days respectively (*p* < 0.001). Baseline characteristics of each group are summarised in Table 1.

**TABLE 1:** Baseline characteristics of patients who underwent diagnostic transperineal prostate biopsy prior to and after government subsidisation of mpMRI of the prostate

	Pre‐MBS subsidisation (*n* = 361)	Post‐MBS subsidisation (*n* = 289)	*p* value
Age (years)	64 (57–69)	65 (59–70)	0.005
PSA (ng/ml)	6.6 (4.8–10.05)	7.6 (5.3–11.4)	0.007
Prostate volume (ml)	40 (30–55)	45 (33–68)	0.002
PSA density (ng/ml/ml)	0.15 (0.10–0.24)	0.15 (0.11–0.27)	0.383
Family history of PCa			0.241
No family history	282 (78.1%)	229 (79.2%)	
Family history	78 (21.6%)	50 (17.3%)	
Unknown	1 (0.03%)	10 (3.5%)	
Biopsy naïve	305 (84.5%)	251 (86.9%)	0.404
DRE			0.198
Benign DRE	225 (62.3%)	164 (56.7%)	
Suspicious DRE	136 (37.7%)	122 (42.2%)	
Not reported	0 (0%)	3 (1.0%)	
mpMRI performed prior to biopsy	131 (36.3%)	224 (77.5%)	<0.001
PI‐RADS			0.131
≤2	30 (22.9%)	44 (19.6%)	
3	27 (20.6%)	39 (17.4%)	
4	52 (39.7%)	88 (39.3%)	
5	22 (16.8%)	53 (23.7%)	
Days between Urology consultation and biopsy	45 (29–69)	63 (40.25–97)	<0.001

*Note*: All results expressed as median (IQR) or *n* (%).

Abbreviations: DRE, digital rectal examination; MBS, Medicare Benefits Schedule; mpMRI, multi‐parametric magnetic resonance imaging; PCa, prostate cancer; PI‐RADS, Prostate Imaging – Reporting and Data System; PSA, prostate‐specific antigen.

One hundred and thirty‐one of 361 patients (36.3%) in the pre‐subsidisation cohort had a pre‐biopsy mpMRI compared with 251 of 289 patients (77.5%) in the post‐MBS subsidisation group (*p* < 0.001). The distribution of PI‐RADS scores was similar between the two groups as outlined in Table 1 (*p* = 0.131).

Table 2 illustrates the distribution of histopathology results stratified by PI‐RADS score. Seventy‐four patients had a PI‐RADS score ≤ 2. Sixty‐four of these patients had no cancer or low‐risk disease, giving mpMRI a negative predictive value (NPV) of 86.5% for clinically significant disease.

**TABLE 2:** Histopathological results of all patients undergoing mpMRI prior to biopsy, stratified by PI‐RADS score

	Total	Negative biopsy	Grade Group 1	Grade Group 2	Grade Group 3	Grade Group 4	Grade Group 5	Total PCa	Total Grade Group ≥ 2
PI‐RADS ≤ 2	74	48 (64.9%)	16 (21.6%)	7 (9.4%)	3 (4.1%)	0 (0%)	0 (0%)	26 (35.1%)	10 (13.5%)
PI‐RADS 3	66	35 (53.0%)	14 (21.2%)	11 (16.7%)	4 (6.1%)	0 (0%)	2 (3.0%)	31 (47.0%)	17 (25.8%)
PI‐RADS 4	140	40 (28.6%)	36 (25.7%)	33 (23.6%)	16 (11.4%)	9 (6.4%)	6 (4.3%)	100 (71.4%)	64 (45.7%)
PI‐RADS 5	75	7 (9.3%)	3 (4.0%)	21 (28.0%)	17 (22.7%)	7 (9.3%)	20 (26.7%)	68 (90.7%)	65 (86.7%)
Total	355	130 (36.6%)	69 (19.4%)	72 (20.3%)	40 (11.3%)	16 (4.5%)	28 (7.9%)	225 (63.4%)	156 (44.0%)

Abbreviations: mpMRI, multi‐parametric magnetic resonance imaging; PCa, prostate cancer; PI‐RADS, Prostate Imaging – Reporting and Data System.

Following mpMRI subsidisation, there was a decrease in the number of negative prostate biopsies. 215 of 361 (59.6%) patients in the pre‐MBS subsidisation harboured PCa, compared with 197 out of 289 patients (68.2%) in the post‐MBS subsidisation group (*p* = 0.024). The rate of clinically significant PCa was higher in the post‐subsidisation cohort compared with the pre‐subsidisation cohort (49.5% vs. 39.1% respectively, *p* = 0.008). There was no significant difference seen in the rate of low‐risk disease detected in both groups with 18.7% of patients in the post‐subsidisation group and 20.5% of patients in the pre‐subsidisation group having Grade Group 1 disease (*p* = 0.563). The distribution of histopathology results is further delineated in Table 3.

**TABLE 3:** Histopathological results of patients who underwent diagnostic transperineal prostate biopsy prior to and after government subsidisation of mpMRI of the prostate

	Pre‐MBS subsidisation (*n* = 361)	Post‐MBS subsidisation (*n* = 289)	*p* value
Positive for prostate cancer	215 (59.6%)	197 (68.2%)	0.024
Grade Group 1	74 (20.5%)	54 (18.7%)	0.563
Grade Group ≥ 2	141 (39.1%)	143 (49.5%)	0.008

*Note*: All results expressed as *n* (%).

On multivariate logistic regression analysis for predicting Grade Group ≥ 2 disease, age (odds ratio [OR], 1.045, *p* = 0.25), suspicious DRE (OR, 3.923, *p* < 0.001), PI‐RADS ≥ 3 (OR, 4.142, *p* < 0.001) and PSA density (OR 2.081 for every 0.1 unit increase, *p* < 0.001) were significant predictors of Grade Group ≥ 2 PCa. These results are further summarised in Table 4.

**TABLE 4:** Multivariate logistic regression analysis of predictive variables of Grade Group ≥ 2 prostate cancer

Characteristic	OR	95% CI	*p* value
Age	1.045	1.006–1.085	0.25
Suspicious DRE	3.923	2.252–6.836	<0.001
PSA density (for every 0.1 unit increase)	4.142	1.909–8.987	<0.001
PI‐RADS ≥ 3	2.081	1.583–2.734	<0.001

Abbreviations: CI, confidence interval; DRE, digital rectal examination; OR, odds ratio; PI‐RADS, Prostate Imaging – Reporting and Data System; PSA, prostate‐specific antigen.

## 4 DISCUSSION

The selection of patients for prostate biopsy remains a topic of some controversy with some variation among major urological guidelines.^14^, ^15^, ^16^ mpMRI has improved the sensitivity for the detection and localisation of Grade Group ≥ 2 PCa with EAU guidelines now recommending mpMRI to be performed prior to biopsy in both biopsy naïve men and patients with previous negative biopsies.^5^, ^6^, ^14^, ^17^ Our study has identified that the introduction of subsidised prostate mpMRI in Australia has resulted in an increase in the proportion of patients with pre‐biopsy mpMRI and has improved rates of diagnosis of PCa, in particular, clinically significant cancer.

The implementation of government subsidised mpMRI has enhanced the diagnostic quality of prostate biopsies. In the post‐subsidisation cohort, there was an increase in biopsies yielding clinically significant PCa (from 39.1% to 49.5% [*p* = 0.008]) without any change in rates of diagnosis of low‐risk disease. Our improved detection rates are similar to previous clinical trials utilising mpMRI in prostate biopsy. Porpiglia et al found pre‐biopsy mpMRI improved the detection rate of PCa of any grade from 29.5% to 50.5% and the detection of clinically significant cancer from 18.1% to 43.9%.^18^ In the PRECISION study, mpMRI targeted biopsies were compared to transrectal biopsies and found to detect a higher rate of clinically significant cancer (38% compared with 26%) and fewer low‐risk PCa (9% compared with 22%).^6^ A recent Cochrane Review also suggested using an mpMRI driven biopsy pathway increased the detection rate of Grade Group ≥ 2 PCa by 12% compared with systematic biopsy.^17^ In our study, the wider availability and uptake of mpMRI following subsidisation has predictably improved the yield of diagnostic transperineal biopsy.

Although a suspicious mpMRI reaffirms the indication for biopsy, clinical decision making following a negative mpMRI is less clear.^19^ The EAU guidelines recommend shared decision making after a negative mpMRI, suggesting omission of biopsy in patients with low suspicion of PCa and systematic biopsy in high risk patients.^14^ The choice to biopsy patients with PI‐RADS ≤ 2 in our institution was based on shared clinician and patient decision making. In our study, mpMRI had an NPV of 86.5% for excluding Grade Group ≥ 2 PCa. In comparison, the ERSPC cohort had an NPV of 92.8%, whereas the PROMIS study demonstrated an NPV of 76% when also excluding Grade Group ≥ 2 disease.^5^, ^20^ Similarly, Hansen et al found an NPV of 80% when utilising MRI alone and 91% when combined with a PSA density of <0.1 ng/ml/ml.^21^

Obtaining an mpMRI prior to biopsy influenced the time to prostate biopsy. In our study, following the introduction of MBS subsidised mpMRI funding, there was an increase of 18 days (*p* < 0.001) from initial Urology clinic review to biopsy. Although statistically significant, given the relatively long natural history of PCa, this delay is less significant clinically and would not preclude the utilisation of mpMRI to optimise decision making.^22^

Although our institution's experience with the increased accessibility of mpMRIs has seen improved selection of patients for transperineal prostate biopsy, we identified several potential areas of improvement. Patients were commonly referred following a single raised PSA. As the Australian criteria for subsidised mpMRI requires two PSA results within the space of 1–3 months, patients were usually required to have a further PSA before a subsequent review in order to qualify for a subsidised mpMRI.^11^ This could potentially be streamlined by raising familiarity among primary care doctors regarding the criteria for mpMRI or by triaging referrals and requesting repeat PSA testing prior to initial review. Furthermore, as demonstrated in our study, pre‐biopsy mpMRI can delay time to biopsy. In patients with grossly elevated PSA or malignant DRE where a negative mpMRI would not preclude biopsy, it may be argued that these patients should continue immediately to biopsy without mpMRI if an extended delay is likely. These changes may reduce the burden of repeat appointments on the public outpatient system and further improve the accessibility and timeliness of mpMRI for patients with suspected PCa.

This study was limited by its retrospective design and single institution experience. Despite being a retrospective study, our dataset was largely complete. Furthermore, although subsidised mpMRI was available from 1 July 2018, it is likely that there was an adjustment period after its introduction where there was reduced uptake. As it was unclear what the duration of this period was, we stratified our cohorts based on the introduction date, accepting that the effects of mpMRI subsidisation demonstrated in this study are, consequently, possibly underestimated.

In conclusion, the recent introduction of a government subsidy for mpMRI of the prostate as a public health policy has correlated with increased utilisation of pre‐biopsy imaging. This has enabled more appropriate patient selection for biopsy and reduced the rate of negative biopsies. However, we acknowledge that the benefits of PCa treatment in Grade Group ≥ 2 disease are based on data from a pre‐mpMRI era and more contemporary studies may be required to explore the benefits of treatment, particularly in patients diagnosed with low volume Grade Group ≥ 2 disease in the mpMRI era. This study demonstrates how funding models have the capacity to significantly impact patient care and how clinicians will rapidly adjust care delivery based on affordability of care for patients. Future studies may aim to perform a cost–benefit analysis of mpMRI subsidisation to evaluate whether similar models could be introduced in a cost‐effective manner in alternate healthcare systems elsewhere.

## CONFLICT OF INTEREST

The authors declare that they have no conflict of interest.

## AUTHOR CONTRIBUTIONS

**Gavin Wei:** data collection; analysis; manuscript production. **Fairleigh Reeves:** data collection; manuscript revision. **Marlon Perera:** data collection; analysis; manuscript production. **Brian D. Kelly:** data collection; manuscript revision. **Stephen Esler:** supervision; manuscript revision. **Damien Bolton:** supervision; manuscript revision. **Greg Jack:** supervision; manuscript revision.

## Supporting information


**Table S1.** Eligibility criteria for MBS subsidised mpMRI^11^
