# Supplementary material for: The impact of health‐policy‐driven subsidisation of prostate magnetic resonance imaging on transperineal prostate biopsy practice and outcomes
Source: BJUI Compass. 2022 Feb 11;3(4):304–9. doi: 10.1002/bco2.140 (PMC9231672; doi:10.1002/bco2.140)
Supplement: Supplementary file 1 — Table S1. Eligibility criteria for MBS subsidised mpMRI11 [file BCO2-3-304-s001.docx]

Supplementary Table 1. Eligibility criteria for MBS subsidised mpMRI^11^

| The patient must be suspected of having PCa based on:   1. a DRE which was suspicious for PCa; or 2. in a person aged less than 70 years, at least two PSA tests performed within an interval of 1 - 3 months of greater than 3.0 ng/ml, and a free/total PSA ratio less than 25% or the repeat PSA exceeding 5.5 ng/ml; or 3. in a person aged less than 70 years, whose risk of developing PCa based on family history is at least double the average risk, at least two PSA tests performed within an interval of 1 - 3 months of greater than 2.0 ng/ml, and a free/total PSA ratio less than 25%; or 4. in a person aged 70 years or older, at least two PSA tests performed within an interval of 1- 3 months of greater than 5.5 ng/ml and a free/total PSA ratio less than 25% |
| --- |
